# 686. Management and Outcomes of Patients Referred to a Specialty Clinic for *C. difficile* Infection

**DOI:** 10.1093/ofid/ofad500.748

**Published:** 2023-11-27

**Authors:** Aaron Hunt, Larry H Danziger, Stuart Johnson, Andrew M Skinner

**Affiliations:** UIC College of Pharmacy, Schaumburg, Illinois; University of Illinois at Chicago, Chicago, IL; Hines VA Hospital and Loyola University Medical Center, Hines, Illinois; University of Utah, Holladay, Utah

## Abstract

**Background:**

*Clostridioides difficile* infection (CDI) poses an urgent threat in the United States with an estimated 223,900 cases of hospitalization and 12,800 estimated deaths in 2017. CDI manifestations range from diarrhea, pseudomembranous colitis, to fulminant colitis. Due to spores that are unaffected by current antimicrobial therapies, recurrence remains a common challenge. Patients with 2 or more episodes have an estimated 40-65% rate of recurrence. We reviewed the management and outcomes of patients referred to our specialty CDI clinic.

**Methods:**

A retrospective chart review was conducted on all new patients presenting to the Loyola University Medical Center CDI clinic between 01/01/2021 and 12/31/2021. Each chart was reviewed for demographic information, comorbidities that may confound diagnosis, and characteristics that place the patient at high risk of recurrent CDI. Visits were characterized by the number of prior CDI episodes, timeline of recurrences, and prior therapies used. 30- and 60-day symptom recurrence were assessed.

**Results:**

A total of 48 new patients presented to the CDI clinic. The median age was 63 years, 64.5% were white, and 64.5% were female. Patients presenting with recurrent CDI accounted for 79% of cases (38/48), with 48% (13/48) of patients having two or more recurrences. The most common decision for initial management was to not give further antimicrobials and monitor for symptoms (35%). 12% of patients only required monitoring while 23% of patients had a questionable CDI diagnosis and were referred elsewhere for further evaluation. For the 30 patients who received treatment, vancomycin or fidaxomicin tapers were selected 73% (22/30) and bezlotoxumab 20% (6/30) of the time. 8.6% (2/23) of patients with a history of two or more CD episodes had recurrence. Overall, patients experienced a subsequent recurrence rate of 12.5% (6/48) and 15% (7/48) at 30 and 60 days, respectively.

**Figure:**
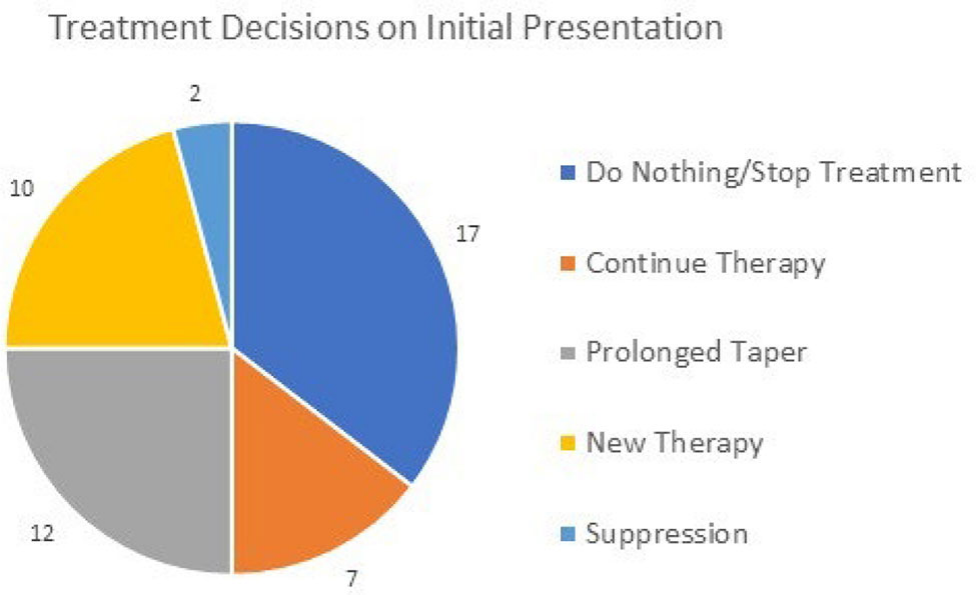

The number of times each management approach was selected for initial patient appointments.

**Figure:**
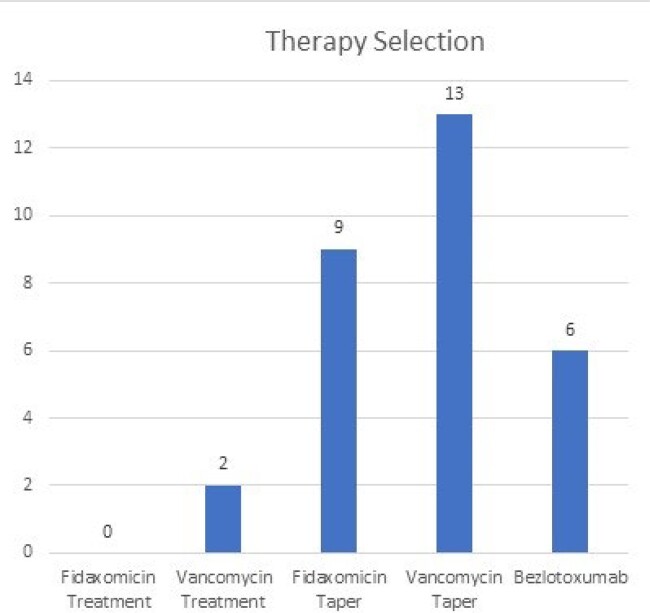

The number of times each treatment regimen was selected when a patient was deemed to need antibiotic therapy.

**Figure:**
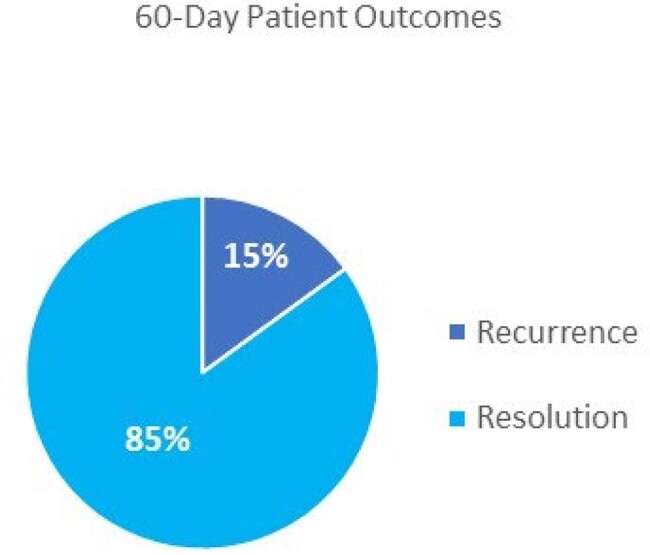

The percentage of patients who had either recurrence or resolution at 60 days after the end of therapy.

**Conclusion:**

CDI is a difficult-to-treat infection with many patients suffering from multiple recurrences. Dedicated outpatient follow-up is important for monitoring therapy and excluding alternate etiologies. Expert management of CDI through a dedicated clinic results in lower recurrence for high-risk patients and spared 35% of patients further antimicrobial exposure.

**Disclosures:**

**Larry H. Danziger, PharmD**, Ferring Pharmacetuicals: Advisor/Consultant **Andrew M. Skinner, MD**, Academy for Continued Healthcare Learning: Honoraria|American Society of Healthcare Pharmacists: Honoraria|Ferring Pharmaceuticals: Honoraria|MJH Life Sciences: Honoraria

